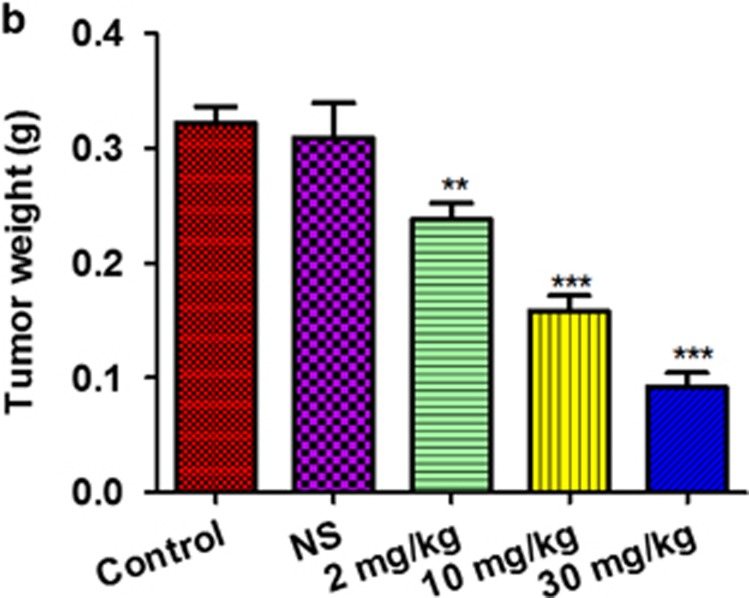# A natural food sweetener with anti-pancreatic cancer properties

**DOI:** 10.1038/oncsis.2017.19

**Published:** 2017-04-10

**Authors:** C Liu, L-H Dai, D-Q Dou, L-Q Ma, Y-X Sun

**Correction to:** *Oncogenesis* (2016) **5**, e217; doi:10.1038/oncsis.2016.28; published online 11 April 2016

Since the publication of the above article, the authors have realized that Figure 4b was incorrect. The corrected figure is shown below.

The following corrections are also required in the Results section on page 3, line 3 from the bottom:

The overall tumor weight in the control group was 0.322±0.014 g, whereas it was only 0.092±0.018 g in the mice that had received the high-dose mogroside V treatment.

The authors would like to apologize to the readers for any inconvenience this may have caused.

## Figures and Tables

**Figure 1:**